# Evolutionary Influenced Interaction Pattern as Indicator for the Investigation of Natural Variants Causing Nephrogenic Diabetes Insipidus

**DOI:** 10.1155/2015/641393

**Published:** 2015-05-28

**Authors:** Steffen Grunert, Dirk Labudde

**Affiliations:** Hochschule Mittweida, University of Applied Sciences, Technikumplatz 17, 09648 Mittweida, Germany

## Abstract

The importance of short membrane sequence motifs has been shown in many works and emphasizes the related sequence motif analysis. Together with specific transmembrane helix-helix interactions, the analysis of interacting sequence parts is helpful for understanding the process during membrane protein folding and in retaining the three-dimensional fold. Here we present a simple high-throughput analysis method for deriving mutational information of interacting sequence parts. Applied on aquaporin water channel proteins, our approach supports the analysis of mutational variants within different interacting subsequences and finally the investigation of natural variants which cause diseases like, for example, nephrogenic diabetes insipidus. In this work we demonstrate a simple method for massive membrane protein data analysis. As shown, the presented in silico analyses provide information about interacting sequence parts which are constrained by protein evolution. We present a simple graphical visualization medium for the representation of evolutionary influenced interaction pattern pairs (EIPPs) adapted to mutagen investigations of aquaporin-2, a protein whose mutants are involved in the rare endocrine disorder known as nephrogenic diabetes insipidus, and membrane proteins in general. Furthermore, we present a new method to derive new evolutionary variations within EIPPs which can be used for further mutagen laboratory investigations.

## 1. Introduction

**Figure 1:**
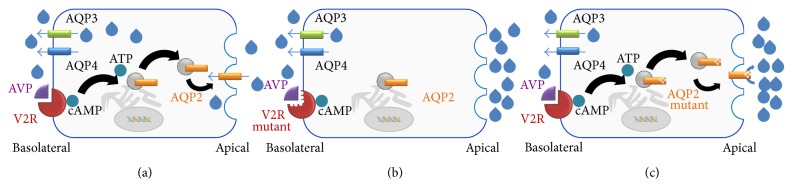
(a) In normally regulated water absorption in kidney cells, the antidiuretic hormone arginine vasopressin (AVP) is released in the pituitary gland, binds to the V2 receptor (V2R), and subsequently induces a series of phosphorylation reactions which lead to the insertion of aquaporin-2 water channels in the apical membrane that allow water molecules to pass the membrane. (b) Genetic mutations in the gene encoding V2R lead to reduced binding affinity and protein stability in V2R. Dysfunctional V2R mutants cause a significantly reduced amount of inserted aquaporin-2 proteins and thus decrease the water flux through the apical membrane. On the other hand, dysfunctional aquaporin-2 mutants decrease the water reabsorption as well (see (c)). Reduced water reabsorption is directly linked to an increased output of highly diluted urine (polyuria) and excessive drinking (polydipsia) which are the most severe symptoms observable in nephrogenic diabetes insipidus patients [12, 18, 20].

Integral membrane proteins are coded by 20–30% of all open reading frames of known genomes [1–3]. As elements in accomplishing numerous molecular processes, that is, signal transduction and passive and active transport of an extensive number of chemical compounds and ions, mutations in genes coding for membrane proteins are often linked to diseases [4]. Despite their biological importance, relatively little is known about folding, functional mechanics, and synthesis of membrane proteins [1]. This is due to experimentally costly and complex procedures, since membrane proteins are difficult to handle in lab experiments [5]. To understand correspondence between genetic mutations and the effects on protein mechanics, the development of novel theoretical approaches is highly demanded. In our work we demonstrate a high-throughput in silico approach for the investigation of the influences of genetic variations within interacting sequence part in membrane proteins, which are directly linked to nephrogenic diabetes insipidus. Nephrogenic diabetes insipidus (NDI) is a disorder which can be acquired as a side effect of surpassing drug taking or which is caused by inherited genetic mutations. Autosomal recessive and dominant inherited NDI are linked to mutations in genes encoding the integral membrane aquaporin-2 water channel [6, 7]. X-linked inheritable NDI is caused by mutations in the gene encoding the AVP type-2 receptor membrane protein (V2R) [8, 9]. In the general population, inherited NDI shows a low prevalence of one case per 20,000–30,000 people [10–12]. Aquaporin-2 water channels and V2R are essential elements in the water reabsorption through the apical cell membrane. This water composes the main part of preurine, a product that results from ultrafiltration in the kidney. The process of water reabsorption from the preurine is essential to ensure the body's fluid balance and is realised by membrane-integrated aquaporin-2 water channels. The insertion of aquaporin-2 into the human kidney cell membrane is triggered by the antidiuretic hormone, which is also referred to as arginine vasopressin (AVP). The AVP blood concentration is regulated by the controlled release of AVP in the pituitary gland which is adapted according to the body's fluid balance. In the process, the binding of AVP to V2R leads to the activation of the receptor. In this state, V2R is able to interact with the guanine nucleotide-binding G(s) subunit alpha [13, 14]. Subsequently, the activation of adenylcyclase 6 takes place, leading to cAMP synthesis and increase of cAMP concentration in the cell plasma [15, 16]. By means of protein kinase A, cAMP triggers the phosphorylation of aquaporin-2 molecules which are stored in cytoplasmic vesicles that have bound to the endoplasmic reticulum. The phosphorylation induces the translocation and fusion of the cytoplasmic vesicles into the plasma membrane and finally leads to the insertion of aquaporin-2 molecules into the apical membrane [17]. Inactive mutants of V2R and aquaporin-2 cause a reduced water reabsorption in the kidneys [18]. Consequences are the typical symptoms of NDI, for example, sensorineural deafness, urinary tract anatomy, ataxia, peripheral neuropathy, mental retardation, psychiatric illness, a daily output of 15–20 L highly dilute (<100 mOsmol/kg) urine (polyuria), and compensatory excessive liquid intake [18–20]. In newborn infants, NDI is characterized by dehydration symptoms, irritability, and poor feeding as well as poor weight gain. A schematic illustration of these molecular coherences is given in Figure 1. The direct inspection of the aquaporin-2 gene as well as the V2 receptor gene (AVPR2) has become accomplishable in clinical practice [21] for differential NDI diagnosis and has been substituting dehydration testing over the last years [18].

## 2. Materials and Methods

As the first step, we want to realise a task which is involved in the prediction of homologue sequence parts within transmembrane *α*-helices. This means that aquaporin specific evolutionary interaction pattern pairs (EIPPs) were generated as described in current work of [22]. In this work, Grunert and Labudde show that the combination of interaction information and sequence motifs with evolutionary variation can be used for 3D structure prediction. They obtained key information from homologue sequences to separate and predict membrane protein structures in the context of interacting pattern and their evolutionary variation. Patterns as motif representatives are investigated for evolutionary covariation. Here, a motif has been described in previous work of [23] and can be written in a generalized, regular expression-like form of XY*n*, where X and Y correspond to amino acids separated by *n* − 1 highly variable positions. Interaction information contributes to detecting interacting pattern with evolutionary background. This means that evolutionary variation at pattern positions was marked as X. Here, different mutation types like that described in [22] may apply at specific X-position. Subsequently, in this work recently published proteins with PDB-Ids: 4nef, 4oj2 were used to transfer family specific EIPPs to these aquaporin-2 representative proteins. For mention, the protein structure (PDB-Id: 4nef, 4oj2) was published by Frick et al. [24] and Vahedi-Faridi et al. [25] Beyond, both protein structures were considered as unknown structures at time of EIPP generation caused by missing Pfam entries. This led to no consideration of both proteins by EIPPs generation. Aquaporin specific EIPPs were derived from known structures of the corresponding PF00230 family. After obtaining of EIPPs, they were employed to generate interaction block schemes (IBSs). Here we try to illustrate that IBSs are useful graphical visualisation media to represent different interacting patterns which distinguish evolutionary. More specifically, we are able to show if a mutation within a pattern has influence on the evolutionary variability of the interacting counterpart. Eventually, IBSs can be used to support the understanding of the three-dimensional fold for the respective interaction partners and the whole protein structure. Moreover, transmembrane helical information was derived from PDBTM [26] for the proteins to be investigated (PDB-Ids: 4nef, 4oj2). Afterwards, EIPPs were applied on helices and sequence similarity of the incurred interacting ranges compared to known structures of the other family members was calculated. For further investigation, mutations occurring in nephrogenic diabetes insipidus patients were aggregated from recently published works [27–34] and registered on sequences of proteins to be investigated. These natural variants of NDI can also be obtained from UniProt (http://www.uniprot.org/uniprot/P41181). Finally, IBSs were applied to similar sequence parts which include NDI mutational effects.

### 2.1. Evolutionary Variations within EIPPs

In this section we describe a method to derive variation at X-positions from evolutionary sequence record. To realise this task, the full unknown seed structure dataset (9641 proteins) of the representative family (PF00230) was derived from Pfam database [35]. Transmembrane helical information was obtained using TMHMM Server v.2.0 [36]. A variety of methods have been developed to predict structural features from sequence, such as *α*-helical membrane-spanning helices and extra/intracellular domains. Basically, TMHMM performs a prediction of intra/extracellular regions and integral membrane helices based on sequence. Beside per-residue predictions TMHMM also lists underlying per-residue assignment probabilities as an indicator of prediction uncertainty. Consequently, helical information was used to apply our derived EIPPs at unknown structures. Here, X-positions were investigated in a closer way when both existing EIPP counterparts were registered in different helices. For the detecting of new evolutionary variations, the amino acid occupancy from unknown structure information was used to compare with amino acids of known structures at specific X-positions. At last, new amino acids at variable X-position were registered and added. Finally, with this method we are able to extend evolutionary information within interacting sequence parts which can be used for further mutational or general investigations of membrane proteins or in this case specifically aquaporin water channel proteins.

## 3. Results and Discussion

**Figure 2:**
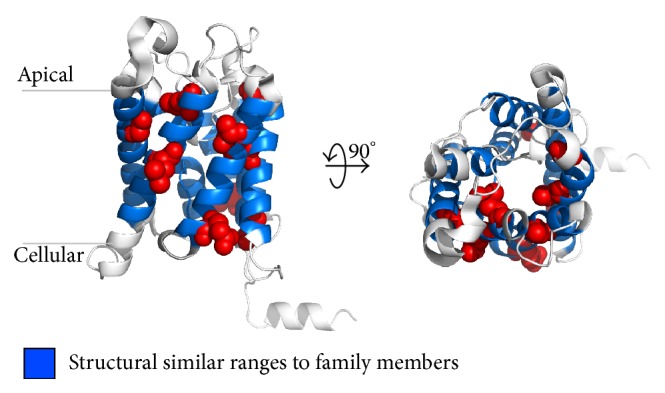
Structural colouring of helical ranges with high similarity to aquaporin family members (PF00230). Side and top-down view of the aquaporin-2 representative protein (PDB-Id: 4nef). Blue coloured cartoon residue ranges are present. These consist of family specific EIPPs which were found in known structures of the given family (PF00230). Red coloured spheres illustrate natural variants derived from UniProt (http://www.uniprot.org/uniprot/P41181) of the protein sequence caused by nephrogenic diabetes insipidus. All figures were rendered with PyMOL.

**Figure 3:**
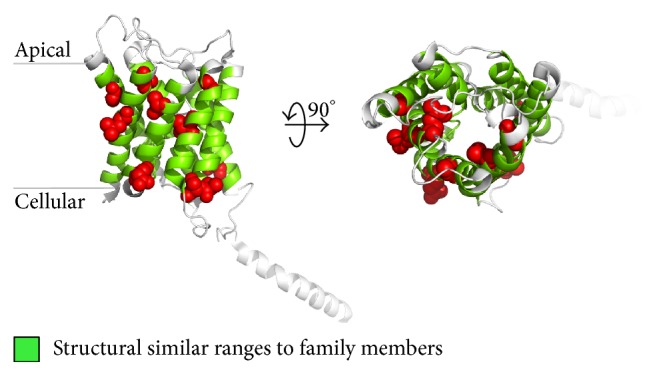
Structural colouring of helical ranges with high similarity to aquaporins (PF00230). Side and top-down view of the aquaporin-2 representative protein (PDB-Id: 4oj2). Green coloured cartoon residue ranges are present. These consist of family specific EIPPs which were found in known structures of the given family (PF00230). Red coloured spheres illustrate natural variants derived from UniProt (http://www.uniprot.org/uniprot/P41181) of the protein sequence caused by nephrogenic diabetes insipidus. All figures were rendered with PyMOL.

Our structure prediction shows, if an unknown structure tends to a family affiliation, family specific EIPPs have to resurface on the protein sequence. Here, EIPPs were derived from known crystal structures of the aquaporin family (PF00230) and marked to *α*-helical structure of recently published aquaporin-2 representative proteins (PDB-Ids: 4nef, 4oj2). As mentioned before, aquaporin-2 representatives have not been considered in the EIPP generation process and make them to transparent unknown structures. Similarity results are shown in Figures 2 and 3 and confirm the already enlightened family affiliation and predicted structures. This means in all TM-helices EIPPs could be found and cover the helical range with up to 100% as listed in Table 1.

**Figure 4:**
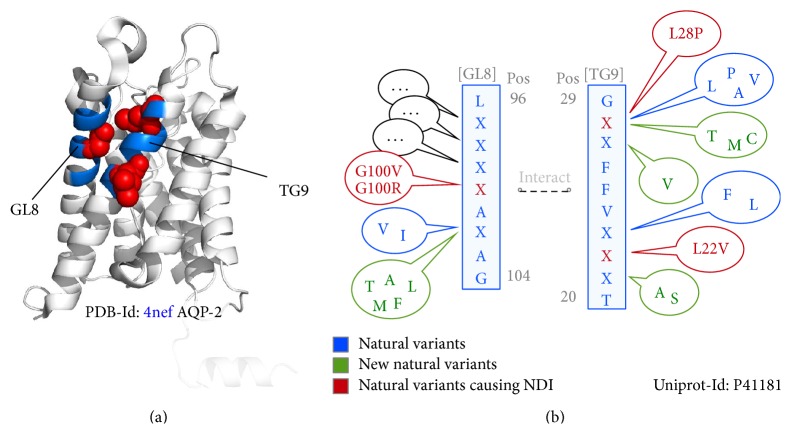
Examples of two spatially interacting subsequences. (a) More specifically, GL8 motif representative pattern (left) interacts with TG9 (right). Both patterns are coloured in blue. Red spheres representing natural variants causing nephrogenic diabetes insipidus (NDI). If we look at these patterns, it is not important to know which pattern position is involved in a spatial interaction. It is more important, that these two pattern build an interacting block. (b) The corresponding interaction block scheme (IBS), with the interacting pattern in blue letters. Red letters illustrating positions causing NDI. All patterns have variable positions. These positions are marked with X. Different coloured bubbles are present and address an X-position with possible natural variants. These are examples of possible occupations, which can be realized by natural variants derived from known structures (blue), natural variants causing NDI (red), or new natural variants derived from unknown structures (green).

Here our prediction results explain the mightiness of EIPPs. On the one hand they provide useful and powerful information to predict *α*-helical structures within the transmembrane environment of homologue membrane proteins. On the other hand, we are able to describe selected interacting areas which are constrained by evolution. To evaluate this assumption, different IBSs were generated and applied to highly conserved interacting sequence parts which were derived from Pfam HMM-logos [35]. One IBS example is shown in Figure 4 which illustrates two interacting patterns. These patterns are part of the aquaporin-2 representative protein with PDB-Id: 4nef. Within an X-positioned pattern, we are able to register evolutionarily designed variable positions. Our IBSs additionally show that an interaction with another pattern takes place. In this work, the goal was not to show which pattern position is involved in spatial interaction but rather to show that two patterns build an interacting block. Figure 4 shows examples of variable positions, which can be occupied by different natural variants. With our IBSs, we can show that an interaction between two blocks is given, when the respective positions are occupied by the amino acids. This implies that a TG9-GL8 interaction is given with Phe23-Ala101 or Phe26 and Ala101 or in the case of NDI caused by Leu28Pro [28], a TG9-GL8 interaction is given with Leu28 and Val102 (red coloured amino acid occupation) at specific positions. Here, IBSs give us a quick overview, that within or across a block evolutionary changes take place. That a destabilizing amino acid substitution is compensated by another position over the evolutionary time scale has been explained in detail in previous work of Morcos et al. [37]. Mutational variation information at specific X-positions can close this gap. This leads to further results in our work, the detection of new X-positioned variations caused by evolution. Here, many variations of new possible amino acid substitutions within different sequence pattern could be obtained. As one example, the TG9 motif representative pattern TLIXVFFXXG is given. For this, X-positions can be occupied with the following amino acids: X3F, X3L, X7G, X8V, X7A, X8P, and X8L, which lead to a final regular expression similar to PROSITE [38–41] pattern syntax TLI[F,L]VFF[G,A][V,A,P,L]G. The evaluation of the VG5 submotif representative pattern VFFGXG shows the flexibility of evolution. Referring to natural variant causing NDI L28P, the fourth position (starting from zero) can be occupied with the following amino acids: X4L, X4P. Here, our method has spawned new amino acids for this position. X4T, X4M, and X4C are able to complete this block which leads to final regular expression VFFG[V,A,P,L,T,M,C]G. Ultimately, this shows a greater variability outside of VG5. For further tasks in genomics or proteomics like protein modelling or mutational investigations, new amino acid replacements can be included. This expands the view on what structural mechanisms could also be possible to realise the three-dimensional fold within the respective sequence part and finally to ensure the protein function.

## 4. Conclusion

In the present work, we have applied a new approach for extracting short, spatially interacting amino acid sequence parts, so-called evolutionary interaction pattern pairs (EEIPs), from known structures of membrane proteins, more specifically aquaporin water channel proteins. Based on EIPPs, structure similarity of recently published aquaporin-2 representative proteins was determined and this in silico analysis confirms the aquaporin family affiliation. EIPPs were obtained and employed to generate interaction block schemes of highly conserved sequence parts annotated with natural variants caused by diabetes insipidus. Newly amino acid variations have been discovered. In our further works we will prove the relevance. In conclusion, it is a fact that disease patterns play an important role in membrane proteins but currently few involved structures are available. Different works have shown mutations on a membrane protein sequence influencing disease patterns. Besides, mutations are used in the diagnosis of biomarkers. However, the application of interaction block schemes can lead to better indicators and this in silico analysis can support laboratory mutagen investigations.

## Figures and Tables

**Table 1:** Structural similar helical ranges of two given aquaporin-2 representatives in relation to aquaporin family members (PF00230). Similarity values describe what percentages are consistent helical ranges which are covered by EIPPs.

PDB-Id: 4nef		PDB-Id: 4oj2	
Helix	Similarity	Helix	Similarity
1	100%	1	100%
2	100%	2	100%
3	79.1%	3	79.3%
4	100%	4	100%
5	94.4%	5	95.2%
6	100%	6	100%
7	100%	7	100%

## References

[1] Marsico A., Labudde D., Sapra T., Muller D. J., Schroeder M. (2007). A novel pattern recognition algorithm to classify membrane protein unfolding pathways with high-throughput single-molecule force spectroscopy. *Bioinformatics*.

[2] Brito G. C., Andrews D. W. (2011). Removing bias against membrane proteins in interaction networks. *BMC Systems Biology*.

[3] Tan S., Hwee T. T., Chung M. C. M. (2008). Membrane proteins and membrane proteomics. *Proteomics*.

[4] Luckey M. (2008). *Membrane Structural Biology: With Biochemical and Biophysical Foundations*.

[5] Sadowski P. G., Groen A. J., Dupree P., Lilley K. S. (2008). Sub-cellular localization of membrane proteins. *Proteomics*.

[6] Deen P. M. T., Verdijk M. A. J., Knoers N. V. A. M. (1994). Requirement of human renal water channel aquaporin-2 for vasopressin-dependent concentration of urine. *Science*.

[7] Mulders S. M., Bichet D. G., Rijss J. P. L. (1998). An aquaporin-2 water channel mutant which causes autosomal dominant nephrogenic diabetes insipidus is retained in the golgi complex. *The Journal of Clinical Investigation*.

[8] van den Ouweland A. M. W., Dreesen J. C. F. M., Verdijk M. (1992). Mutations in the vasopressin type 2 receptor gene (AVPR2) associated with nephrogenic diabetes insipidus. *Nature Genetics*.

[9] Rosenthal W., Seibold A., Antaramian A. (1992). Molecular identification of the gene responsible for congenital nephrogenic diabetes insipidus. *Nature*.

[10] Ananthakrishnan S. (2009). Diabetes insipidus in pregnancy: etiology, eva luation, and management. *Endocrine Practice*.

[11] Krysiak R., Kobielusz-Gembala I., Okopien B. (2010). Recurrent pregnancy-induced diabetes insipidus in a woman with hemochromatosis. *Endocrine Journal*.

[12] Robertson G. L. (1995). Diabetes insipidus. *Endocrinology & Metabolism Clinics of North America*.

[13] Wettschureck N., Offermanns S. (2005). Mammalian G proteins and their cell type specific functions. *Physiological Reviews*.

[14] Milligan G., Kostenis E. (2006). Heterotrimeric G-proteins: a short history. *British Journal of Pharmacology*.

[15] Defer N., Best-Belpomme M., Hanoune J. (2000). Tissue specificity and physiological relevance of various isoforms of adenylyl cyclase. *American Journal of Physiology: Renal Physiology*.

[16] Hanoune J., Pouille Y., Tzavara E. (1997). Adenylyl cyclases: structure, regulation and function in an enzyme superfamily. *Molecular and Cellular Endocrinology*.

[17] Kanehisa M., Goto S. (2000). KEGG: kyoto encyclopedia of genes and genomes. *Nucleic Acids Research*.

[18] Los E. L., Deen P. M. T., Robben J. H. (2010). Potential of nonpeptide (ant)agonists to rescue vasopressin v2 receptor mutants for the treatment of X-linked nephrogenic diabetes insipidus. *Journal of Neuroendocrinology*.

[19] Strom T. M., Hörtnagel K., Hofmann S. (1998). Diabetes insipidus, diabetes mellitus, optic atrophy and deafness (DIDMOAD) caused by mutations in a novel gene (wolframin) coding for a predicted transmembrane protein. *Human Molecular Genetics*.

[20] Birnbaumer M. (2002). V2R structure and diabetes insipidus. *Receptors and Channels*.

[21] Fujiwara T. M., Bichet D. G. (2005). Molecular biology of hereditary diabetes insipidus. *Journal of the American Society of Nephrology*.

[22] Grunert S., Labudde D. The observation of evolutionary interaction pattern pairs in membrane proteins.

[23] Liu Y., Engelman D. M., Gerstein M. (2002). Genomic analysis of membrane protein families: abundance and conserved motifs. *Genome biology*.

[24] Frick A., Eriksson U. K., de Mattia F. (2014). X-ray structure of human aquaporin 2 and its implications for nephrogenic diabetes insipidus and trafficking. *Proceedings of the National Academy of Sciences of the United States of America*.

[25] Vahedi-Faridi A., Lodowski D., Schenk A.

[26] Kozma D., Simon I., Tusnády G. E. (2013). PDBTM: protein data bank of transmembrane proteins after 8 years. *Nucleic Acids Research*.

[27] Canfield M. C., Tamarappoo B. K., Moses A. M., Verkman A. S., Holtzman E. J. (1997). Identification and characterization of aquaporin-2 water channel mutations causing nephrogenic diabetes insipidus with partial vasopressin response. *Human Molecular Genetics*.

[28] Marr N., Bichet D. G., Hoefs S. (2002). Cell-biologic and functional analyses of five new Aquaporin-2 missense mutations that cause recessive nephrogenic diabetes insipidus. *Journal of the American Society of Nephrology*.

[29] Lin S.-H., Bichet D. G., Sasaki S. (2002). Two novel aquaporin-2 mutations responsible for congenital nephrogenic diabetes insipidus in Chinese families. *The Journal of Clinical Endocrinology & Metabolism*.

[30] Carroll P., Al-Mojalli H., Al-Abbad A. (2006). Novel mutations underlying nephrogenic diabetes insipidus in Arab families. *Genetics in Medicine*.

[31] Mulders S. M., Knoers N. V. A. M., van Lieburg A. F. (1997). New mutations in the aqp2 gene in nephrogenic diabetes insipidus resulting in functional but misrouted water channels. *Journal of the American Society of Nephrology*.

[32] Vargas-Poussou R., Forestier L., Dautzenberg M. D., Niaudet P., Déchaux M., Antignac C. (1997). Mutations in the vasopressin v2 receptor and aquaporin-2 genes in 12 families with congenital nephrogenic diabetes insipidus. *Journal of the American Society of Nephrology*.

[33] Goji K., Kuwahara M., Gu Y., Matsuo M., Marumo F., Sasaki S. (1998). Novel mutations in aquaporin-2 gene in female siblings with nephrogenic diabetes insipidus: evidence of disrupted water channel function. *The Journal of Clinical Endocrinology & Metabolism*.

[34] Kuwahara M. (1998). Aquaporin-2, a vasopressin-sensitive water channel, and nephrogenic diabetes insipidus. *Internal Medicine*.

[35] Punta M., Coggill P. C., Eberhardt R. Y. (2012). The Pfam protein families database. *Nucleic Acids Research*.

[36] Krogh A., Larsson B., von Heijne G., Sonnhammer E. L. L. (2001). Predicting transmembrane protein topology with a hidden Markov model: application to complete genomes. *Journal of Molecular Biology*.

[37] Morcos F., Pagnani A., Lunt B. (2011). Direct-coupling analysis of residue coevolution captures native contacts across many protein families. *Proceedings of the National Academy of Sciences of the United States of America*.

[38] Sigrist C. J. A., De Castro E., Cerutti L. (2013). New and continuing developments at PROSITE. *Nucleic Acids Research*.

[39] Sigrist C. J. A., Cerutti L., Hulo N. (2002). Prosite: a documented database using patterns and profiles as motif descriptors. *Briefings in bioinformatics*.

[40] de Castro E., Sigrist C. J. A., Gattiker A. (2006). Scanprosite: detection of prosite signature matches and prorule-associated functional and structural residues in proteins. *Nucleic Acids Research*.

[41] Sigrist C. J. A., de Castro E., Langendijk-Genevaux P. S., Le Saux V., Bairoch A., Hulo N. (2005). ProRule: a new database containing functional and structural information on PROSITE profiles. *Bioinformatics*.

